# Infection with *Acinetobacter baumannii* in an intensive care unit in the Western part of Romania

**DOI:** 10.1186/s12879-016-1399-0

**Published:** 2016-03-08

**Authors:** Voichița Lăzureanu, Mirela Poroșnicu, Ciprian Gândac, Teodora Moisil, Luminița Bădițoiu, Ruxandra Laza, Virgil Musta, Alexandru Crișan, Adelina-Raluca Marinescu

**Affiliations:** University of Medicine and Pharmacy, Timisoara, Romania; Clinical Hospital for Infectious Diseases Dr. Victor Babes, Strada Gheorghe Adam, Nr. 13, Timisoara, Romania

**Keywords:** *Acinetobacter baumannii*, Intensive Care Unit, Mechanical ventilation, Antibiotic resistance, Infection

## Abstract

**Background:**

*Acinetobacter baumannii* is one of the main causes of morbidity and mortality in critical condition patients. The pathogen’s ability to survive under a wide range of environment conditions and to persist for long periods of time on areas represents a frequent cause of endemic infection hotbeds especially in the Intensive Care Unit.

The objectives of the study are: determining the 5-year incidence of *A. baumannii* infection in patients admitted in the ICU which needed mechanical ventilation; the analysis of these cases regarding pathological antecedents; processing the data regarding these cases; gradual analysis of the susceptibility/resistance of isolated *A. baumannii* strains; observing the emergence of *A. baumannii* infection in patients transferred into the ICU.

**Methods:**

We have performed an observational retrospective study regarding the incidence of *Acinetobacter baumannii* infections in the Intensive Care Unit of the Hospital of Infectious Diseases and Pneumophtisiology “Victor Babes” Timisoara, Clinic II Infectious Diseases, during June 2011 – June 2015.

**Results:**

We have identified a high prevalence of *Acinetobacter baumannii* infection, with an average period of 6 days. Bronchial suction was the most common pathological product in the study (90 % of the cases). Resistance to antimicrobials has been determined: the lowest resistance was recorded for ampicillin + sulbactam (81.1 %), and the highest resistance rate was recorded for ceftazidime and imipenem (94.6 % each). When comparing resistance to third generation cephalosporins, the difference was not statistically significant (94.6 % for ceftazidime vs. 86.5 % for cefoperazone, *p* = 0.117). Within the present study we were able to observe a significantly high resistance of the germ to carbapenems, with a good sensitivity to aminoglycosides, and to colistin. Only one strain of *Acinetobacter baumannii* was resistant to all classes of tested antibiotics.

**Conclusions:**

Generally, carbapenems represented the elective treatment in severe infections; however, the number of carbapenem-resistant *Acinetobacter baumannii* strains is growing, dramatically reducing therapeutic options, fact that brings back to our attention reusing colistin, although the administration of this antibiotic has been limited due to new antibiotics classes.

## Background

*Acinetobacter baumannii* is a feared Gram-negative bacillus, non-fermentative, capsulated, aerobic, and ubiquitous, which gains territory in the fight against nosocomial infections, especially in the ventilated patient. The emergence and fast spread of this germ raises signs of difficulty as regards the therapeutic management of critical patients. This bacteria’s pan resistance, achieved through insufficiently elucidated mechanism, places *Acinetobacter baumannii* on the list of potentially lethal pathogenic agents.

In Central Asia and Eastern Europe different levels of resistance have been recorded; antimicrobial resistance was reported as a serious threat in countries such as Belarus, Serbia, Switzerland, the Former Yugoslav Republic of Macedonia and Turkey. Switzerland and northern European countries generally face low antibiotic resistance and high awareness, but a recent report has identifies relatively high resistance to carbapenems in *Acinetobacter* spp. [1].

Objectives: determining the 5-year incidence of *A. baumannii* infection in patients admitted in the ICU which needed mechanical ventilation; the analysis of these cases regarding pathological antecedents; processing the data regarding these cases, with several measures for limiting the transmission of nosocomial infections, especially in patients that require mechanical ventilation; gradual analysis of the susceptibility/resistance of isolated *A. baumannii* strains; observing the emergence of *A. baumannii* infection in patients transferred into the ICU.

## Methods

We have performed an observational retrospective study regarding the incidence of *Acinetobacter baumannii* infections in the Intensive Care Unit (ICU) of the Hospital of Infectious Diseases and Pneumophtisiology “Dr. Victor Babes” Timisoara, Clinic II Infectious Diseases, during June 2011 – June 2015. The database has been processed with Excel, Microsoft Office 2010 and SPSS Statistics for Windows (version 22.0, IBM Corp, Armonk, NY, USA). The whole database has been structurally built in Excel, Microsoft Office 2010, using its statistic function. The vertical histograms were used for comparisons and the sectional graphics were used to report certain parts to a whole. The emergence’s evolution was built using linear graphics. The Z test was performed to ascertain differences between proportions. The statistical tests were two-sided, and the value for statistical significance was set at *p* ≤ 0.05.

All data come from patients admitted in the local ICU, which is composed of 5 beds, 1 attending doctor, 5 assistants and one nurse. Our ICU presents a low patient flow which require, because of present or ulteriorly “gained” pathologies, a longer hospitalization (with an average of 30 patients per year).

There were no specific criteria for including or excluding the patients in the study, basically all mechanically ventilated patients with *Acinetobacter baumannii* infection were included in the study.

All the patients transferred to the ICU required mechanical ventilation due to severe respiratory failure acquired (patients known as bronchitic or asthmatic). Bronchial suction was collected through regular methods by using the sterile suction probe, in sterile recipients, by medical assistants and the senior doctor. Hemocultures were processed in the Bactec system (Beckton-Dickinson, Franklin Lakes, NJ, USA), the rest of the samples have been processed through conventional methods. Strain identification was performed through classical bio-chemical methods and through the Vitek 2 Compact system (BioMerieux, Marcy-L'Étoile, France). Antibiotic testing was performed through the Vitek 2 Compact system and also through the Kirby-Bauer method, and results were interpreted according to the guidelines elaborated by CLSI (Clinical and Laboratory Standard Institute) [2].

## Results and discussions

Between June 2011 and June 2015 we have investigated 37 patients admitted in the ICU coming from the 3 clinics compiling the Hospital for Infectious Diseases "Dr. Victor Babeș" Timișoara: Pneumology, Clinic I and Clinic II of Infectious Diseases, respectively.

87 % of the patients presented chronic obstructive pulmonary disease, the reason why various anterior antibiotic regimens had been administered, like cephalosporins, cephalosporins + quinolones, carbapenems, fact which could represent an ulteriorly gained antibiotic resistance motif. Only 13 % of the patients introduced in the study had not received prior antibiotic treatment.

All patients within the present study had been transferred to the ICU from the very first hospitalization hours because of the need for mechanical ventilation. Bronchitic or asthmatiform pathology corroborated with pancytopenic status; deterioration of the hemodynamic status and of the respiratory parameters had imposed this therapeutic behavior.

**Fig. 1:**
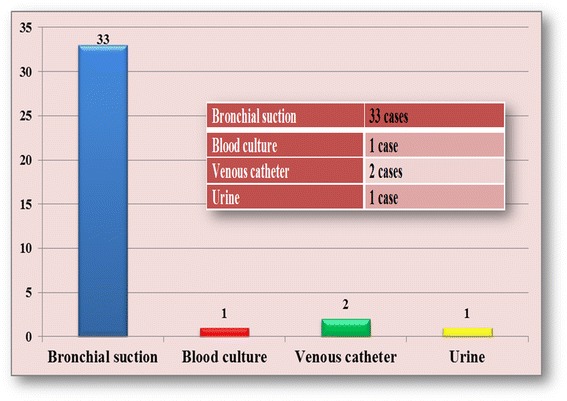
Clinical samples used for the isolation of *Acinetobacter baumannii* strains included in the study

The bacterial strains had been isolated from the following type of clinical samples: bronchial suctioning (33), hemoculture (1), venous catheter (2), urine (1) (Fig. 1).

The present signs and symptoms such as high fever, aggravation of general condition, modification of bronchic secretion, microbian flora encountered in the urine summary had determined a revision of the case towards other diagnostic behaviors, with the request of additional investigations, adapted to each case. The catheter blood culture was positive with a negative peripheral blood culture. Prior to the admission into the ICU, the patient with positive hemoculture was bedsore, having been hospitalized multiple times, fact that could represent an access gate for germs, especially the ubiquitous ones.

The timespan from admission to the ICU to *A. baumannii* detection was, on average, 6 days. The average hospitalization period of patients in the ICU was 28 days. None of the patients had suffered surgical interventions or invasive procedures prior to the admission in the therapy section.

Data obtained after analyzing resistance to *A. baumannii* strains isolated in the bacteriology lab showed that this bacteria’s antibiotic resistance is high. Multidrug resistant bacteria are described as being resistant to at least one agent in 3 or more antibiotic categories. One of the possible causes of the *Acinetobacter baumannii* resistance is non-judicious use of large spectrum antibiotics.

**Fig. 2:**
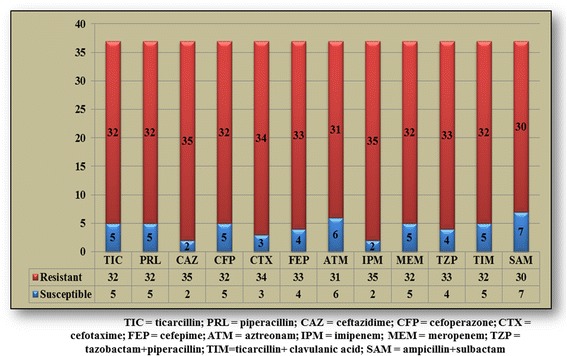
Beta-lactam resistance of bacterial strains included in the study

In the past, the species of *Acinetobacter* showed resistance only to penicillin G, today this resistance is met only in “non-*baumannii*” species. Aminopenicillin, aminopenicillin + clavulanic acid, 1^st^ and 2^nd^ generation cephalosporins resistance defines the wild phenotype [3]. Beta-lactam susceptibility in the strains from our study is reproduced in Fig. 2. An explanation to resistance growth can be provided by the emergence of *Acinetobacter* strains producing beta-lactamases.

Carbapenem resistance in *A. baumannii* has increasingly been reported in European countries. The prevalence of carbapenem resistance appears to be highest in Turkey, Greece, Italy, Spain, and England, and increasing in Eastern Europe [4].

In our study, the lowest resistance was recorded for ampicillin + sulbactam (81.1 %), and the highest resistance rate was recorded for ceftazidime and imipenem (94.6 % each). Resistance to third generation cephalosporins such as ceftazidime was higher than that for fourth generation cephalosporins such as cefepime (94.6 % vs. 89.2 %), *p* = 0.198 (Table 1). Conversely, resistance to third generation cephalosporins such as cefoperazone was lower than that for fourth generation cephalosporins such as cefepime (86.5 % vs. 89.2 %), *p* = 0.359. When comparing the two above mentioned third generation cephalosporins, the difference was not statistically significant (94.6 % for ceftazidime vs. 86.5 % for cefoperazone, *p* = 0.117). One of the explanations could be the fact that generally ceftazidime is used more than cefoperazone in *A. baumannii* infections and this could lead in time, alongside with other aspects, to the development of resistance. The lack of statistical significance is probably due on the one hand to the low number of tested strains, and on the other hand to the overall increased resistance to all tested antibiotics. Carbapenems, particularly meropenem, represented the preferred treatment in these severe infections; nevertheless, the number of carbapenem-resistant *Acinetobacter baumannii* strains is growing, fact that reduces dramatically the therapeutic options, particularly since it is well known that *Acinetobacter baumannii* owns a gene class for beta-lactamases that can provide resistance to carbapenems, the expression of these genes being controlled by mobile promoters. There have also been discovered other resistance genes, including class C chromosomal beta-lactamase gene which provides resistance to cephalosporins; these are controlled in the same way [5].

**Table 1:** Statistical analysis of resistance to antibiotics in the isolated strains of *Acinetobacter baumannii*

Antibiotic agent 1	Strains resistant to antibiotic 1, n/N (%)	Antibiotic agent 2	Strains resistant to antibiotic 2, n/N (%)	*p* value	Z-score
imipenem	35/37 (94.6)	meropenem	32/37 (86.5)	0.117	1.1917
ampicillin + sulbactam	30/37 (81.1)	ticarcillin	32/37 (86.5)	0.264	−0.6308
ceftazidime	35/37 (94.6)	cefoperazone	32/37 (86.5)	0.117	1.1917
ceftazidime	35/37 (94.6)	cefepime	33/37 (89.2)	0.198	0.8518
cefoperazone	32/37 (86.5)	cefepime	33/37 (89.2)	0.359	−0.3557

**Fig. 3:**
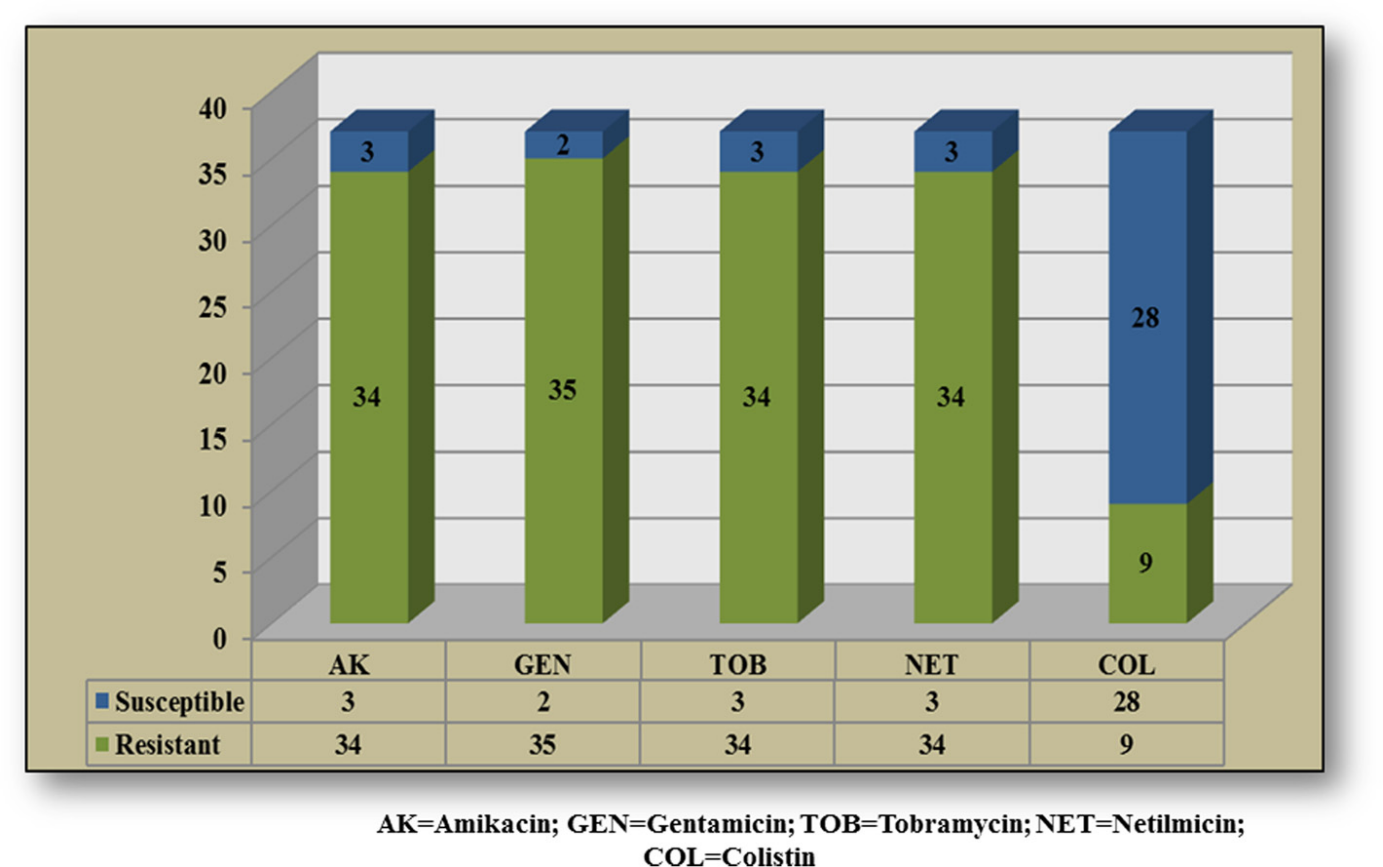
Aminoglycosides and colistin resistance of bacterial strains included in the study

**Fig. 4:**
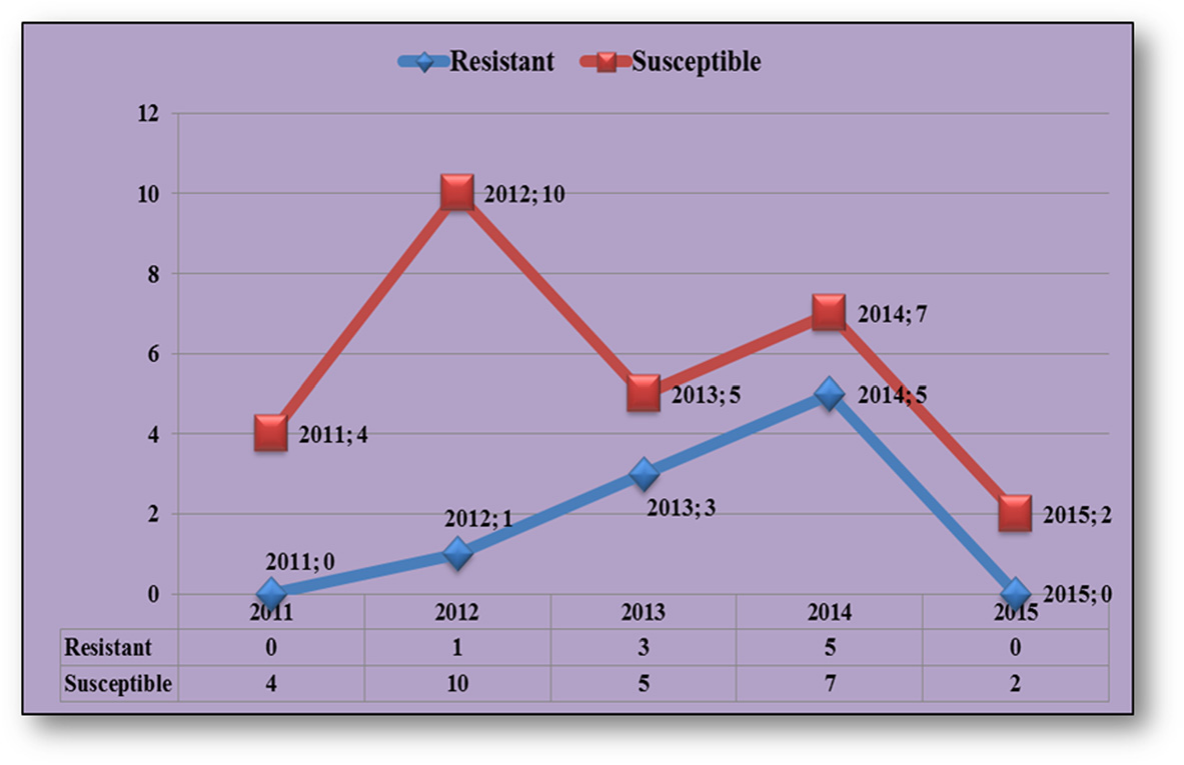
Yearly susceptibility to colistin of bacterial strains included in the study

Within the last 30 years, the use of colistin has been limited due to toxicity issues, as compared to new antibiotic classes. However, the use of colistin has been revaluated in treating multidrug-resistant *Acinetobacter baumannii* strains, susceptible to colistin [6]. Retrospective studies and case reports support in specialty literature colistin treatment for patients with severe central nervous system infection [7, 8]. The doses need to be modified for patients with kidney failure. Within the study we can observe a good susceptibility of the to colistin as compared to the other tested antimicrobials – Fig. 3. The yearly susceptibility of *Acinetobacter baumannii* to aminoglycosides and colistin shows the metamorphosis power of the germ, while maintaining a satisfying susceptibility to colistin – Fig. 4.

Fluoroquinolones resistance is given by the modification of the targets; only 10 % of the tested strains in this study proved susceptible to tested fluoroquinolones.

According to the antibiograms, the patients were treated with colistin in 28 cases and in 9 cases other therapeutic regimens were administered: penicillins active on Gram-negative agents (piperacillin, ticarcillin), cephalosporins (ceftazidime), associations of cephalosporins with aminoglycosides or quinolones, and combinations of 2 antibiotics ticarcillin + tazobactam, ticarcillin + clavulanic acid, ampicillin + sulbactam).

A single strains from our study developed pan resistance (resistance to all the categories of tested antibiotics).

There is partial follow-up data available: 17 patients died, but they were known with significant medical history and multiple comorbidities such as acute myocardial infarction, pulmonary thromboembolism, or stroke. The other 20 patients were transferred back to the previous wards (8 patients are still being followed up within Pneumology clinics).

A limitation of this study can also be represented by the impossibility of performing genetic tests to ascertain the complex mechanisms of resistance to antibiotics, due to technical reasons of equipment of the bacteriology laboratory. The study will also have to be extended and completed with other cases, being known the fact that the higher the number of cases, the more complex and useful the study is in elaborating concrete measures of practical utility.

## Conclusions

*Acinetobacter baumannii* remains one of the most feared agents in nosocomial infections, due to its ability to acquire quicker and tougher resistance mechanisms as compared with other Gram-negative bacteria. Our study has shown that a large number of *A. baumannii* strains were resistant to the tested antibiotics. The lowest resistance was recorded for ampicillin + sulbactam and the highest resistance rate was recorded for ceftazidime and imipenem In these cases, colistin may remain the only active antibiotic and it becomes the preferred antibiotic in treating these infections.

We consider that *A. baumannii* infection can be considered a ubiquitous nosocomial infection with long-term implications in poly-hospitalized patients.

## Abbreviations

ICU: Intensive Care Unit
MDR: Multidrug resistance
